# Embryonic Development and Rates of Metabolic Activity in Early and Late Hatching Eggs of the Major Malaria Vector *Anopheles gambiae*

**DOI:** 10.1371/journal.pone.0114381

**Published:** 2014-12-05

**Authors:** Maria L. Kaiser, Frances D. Duncan, Basil D. Brooke

**Affiliations:** 1 Wits Research Institute for Malaria, School of Pathology, Faculty of Health Sciences, University of the Witwatersrand, Johannesburg, South Africa; 2 Centre for Opportunistic, Tropical & Hospital Infections, National Institute for Communicable Diseases, NHLS, Johannesburg, South Africa; 3 School of Animal, Plant and Environmental Sciences, University of the Witwatersrand, Johannesburg, South Africa; Kansas State University, United States of America

## Abstract

*Anopheles gambiae* eggs generally hatch at the completion of embryo development; two-three days post oviposition. However, staggered or delayed hatching has been observed whereby a single batch of eggs shows marked variation in time-to-hatch, with some eggs hatching 18 days post oviposition or later. The mechanism enabling delayed hatch has not been clearly elucidated but is likely mediated by environmental and genetic factors that either induce diapause or slow embryo development. This study aimed to compare metabolic activity and embryonic development between eggs collected from sub-colonies of the baseline *Anopheles gambiae* GAH colony previously selected for early or late time-to-hatch. Egg batches from early and late hatch sub-colonies as well as from the baseline colony were monitored for hatching. For both time-to-hatch selected sub-colonies and the baseline colony the majority of eggs hatched on day two post oviposition. Nevertheless, eggs produced by the late hatch sub-colony showed a significantly longer mean time to hatch than those produced by the early hatch sub-colony. The overall proportions that hatched were similar for all egg batches. CO_2_ output between eggs from early and late hatch sub-colonies showed significant differences only at 3 and 7 days post oviposition where eggs from the early hatch and the late hatch sub-colony were more metabolically active, respectively. No qualitative differences were observed in embryo development between the sub-colonies. It is concluded that all viable embryos develop to maturity at the same rate and that a small proportion then enter a state of diapause enabling them to hatch later. As it has previously been shown that it is possible to at least partially select for late hatch, this characteristic is likely to involve genetic as well as environmental factors. Delayed hatching in *An. gambiae* is likely an adaptation to maximise reproductive output despite the increased risk of desiccation in an unstable aquatic environment.

## Introduction

*Anopheles gambiae sensu stricto* is the nominal member of the *Anopheles gambiae* species complex. There are currently eight recognized members in this complex [1] of which three: *An. coluzzii* Coetzee and Wilkerson sp.n (previously *An. gambiae* M form), *An. gambiae* Giles (previously *An. gambiae* S form) and *An. arabiensis* Patton, are major African malaria vectors [2], [3].

In *An. gambiae*, intra-species adaptive plasticity that enhances population survival in variable or unpredictable environments also inadvertently maintains malaria parasite transmission. Some of those adaptive traits that likely affect vector capacity include larval development time, adult body size and adult longevity. These are postulated to be under the control of genetic and environmental factors in approximately equal measure in *An. gambiae* under laboratory conditions, although environmental effects are likely of greater importance under natural conditions [4].

An often ignored trait that may affect total development time to adulthood as well as the quality of individuals produced in terms of overall fitness is time-to-hatch. *Anopheles gambiae* are monoandrous and in nature 97% of females mate only once [5]. Oviposition occurs once a female has consumed enough blood to mature her eggs and has selected a suitable oviposition site. The selection of oviposition sites by anopheline females is influenced by factors such as water type and land cover type, and the larval site selected also affects egg hatchability [6], [7]. Larval habitats typically selected by *Anopheles gambiae* tend to be small, temporary water bodies such as water-filled tyre tracks and hoof prints [8]. They can thus be described as unstable because they are prone to periodic desiccation and flooding.

As eggs are fertilized during oviposition by sperm stored in the female's spermatheca after mating, a single batch of eggs oviposited in one sitting is fertilized at approximately the same time and development of the embryo commences once the eggs are oviposited [9]. Eggs normally hatch once embryogenesis is complete which is within two to three days post oviposition in optimal conditions in *An. gambiae*. However, some eggs are able to hatch two weeks or more after oviposition [10], [11] increasing their risk of desiccation even though anopheline eggs normally do not cope well with desiccation. Hatching in mosquito eggs has been shown to be affected by several other factors including temperature [12], vibrations [13], a drop in O_2_ concentration [14] (which may be caused by several factors) and the presence of bacteria [15]- although the majority of this work has been done on *Aedes* species.

Anopheline eggs kept at least slightly moist have previously been shown to hatch up to 18 days after oviposition [16]. Huang *et al.* [17] showed that *An. gambiae* eggs kept in water or on damp soil can survive even at high temperatures while eggs kept on dry soil for more than a few hours are unlikely to hatch, although it must be noted that this observation was also linked to temperatures that exceeded 41°C.

Beier *et al* [18] and Shililu *et al* [19] independently studied hatching of *An. gambiae* s.l eggs on soil in Kenya. Beier *et al* [18] collected dry soil samples from animal hoofprint depressions, the edges of temporary and permanent pools, and stream beds. Two to five days after flooding these samples in water, first instar larvae were observed. No larvae were seen in soil samples taken from stream banks or ploughed fields which are unlikely sites for oviposition. Shililu *et al* [19] found that eggs remained viable for up to 15 days on moist soil and that hatch was influenced by the rate of soil drying, the type of soil and egg age. Minakawa *et al* [20], also in western Kenya, studied oviposition site preference in *An. gambiae* and found that the order of preference was flooded soil, moist soil, dry soil and a blank dish. Mosquitoes by far preferred the flooded soil. However, when dry season conditions were simulated by providing only moist soil, dry soil and an empty dish, the majority of eggs were laid on the moist soil substrate. The authors suggest that this is a strategy to maintain populations through the dry season in combination with the knowledge that *An. gambiae* embryos can survive on moist soil for several days. These studies clearly demonstrate the ability of some eggs to survive in a desiccating environment for a few days to more than two weeks. However, Yaro *et al.* [11] quantified the distribution of hatching times in *An. gambiae*, *An. coluzzii* and *An. arabiensis* in different water types, but without desiccation, and found that most (89%) of the eggs hatched early during the second and third days post oviposition, ten percent hatched between days four and seven and one percent hatched after the first week. They also found that eggs that hatched early developed to adulthood faster and produced smaller adults than late hatchers. Kaiser *et al* [21] and Ebrahimi *et al* [22] have confirmed that desiccation is not a requirement for delayed hatching and a proportion of eggs may be programmed to delay hatching or to hatch in response to a different number or intensity of egg disturbance events or hatching triggers (inundation with water, rainfall, agitation). The ability to delay or stagger larval time-to-hatch is therefore likely an adaptive trait that increases reproductive output despite the increased risk of desiccation in an unstable environment [11], [18], [19]. This trait has been extensively studied in flood water mosquitoes (*Aedes* species) which can delay hatching for several months [23], [24]. There is a comparative scarcity of information available for anopheline mosquitoes.

Evidence of a genetic association with staggered larval time-to-hatch in *An. gambiae* is based on time-to-hatch phenotypic selections and cross-mating experiments [21]. This evidence also includes an association between larval time-to-hatch and the assortment of insecticide resistance phenotypes in a laboratory colony of *An. gambiae*, showing that selection for either early or late larval time-to-hatch affects the frequencies of those factors associated with insecticide resistance as a consequence of pleiotropy. Based on these data it is proposed that staggered larval time-to-hatch occurs because a proportion of individuals are genetically predisposed to hatch late which coincidentally offers a broader platform for the selection of insecticide resistance [21].

However, the processes that enable some *An. gambiae* eggs from the same batch to hatch substantially later than others are unknown. The aim of this study was to investigate the mechanism of staggered larval time-to-hatch by examining embryo development and metabolic output in *An. gambiae* eggs drawn from early and late hatching mothers.

## Materials and Methods

### Biological material

The *Anopheles gambiae* colony (GAH) established using wild-caught females from Ahafo, Ghana, in 2006 was used for all experiments. This colony has undergone selections for early and late time time-to-hatch and has previously been described [21]. Cross-mating experiments [21] indicated that time-to-hatch did not follow a Mendelian mode of inheritance although selection for late time-to-hatch increased the proportion of late hatching larvae to 30%. Both sub-colonies, however, still contained early and late time-to-hatch eggs. The mosquitoes were reared in the Botha de Meillon insectary at the Vector Control Research Laboratory of the National Institute for Communicable Diseases, NHLS, Johannesburg, South Africa. Insectary conditions were maintained at approximately 25°C and 75–85% relative humidity with a 12 hour light: dark cycle with 30 minute dawn and dusk transitions. Larvae were provided with ground dog biscuits (BEENO) and yeast. Adult mosquitoes were maintained as per Hunt *et al*. [25] on a ten percent sucrose solution and females received two to three blood meals per week.

### Time-to-hatch monitoring

Eggs from the early and late time-to-hatch selected sub-colonies as well as from the baseline colony from which the sub-colonies were drawn were monitored for time-to-hatch. This was done to determine whether the selections previously described [21] still had an effect on the time-to-hatch phenotype. Experiments were set up in the Botha de Meillon insectary and maintained at standard conditions. Five batches of eggs were monitored each for the baseline colony and the early time-to-hatch selected sub-colony. For the late time-to-hatch selected sub-colony, 7 batches of eggs were used as the egg batches obtained from this colony were smaller because there were fewer egg producing adults. Eggs were monitored daily for time-to-hatch by counting and removing hatchlings.

### Egg collection and storage for egg metabolic rate experiments

At least three batches of eggs obtained from the early and late time-to-hatch selected sub-colonies were collected on different days after allowing a group of females to oviposit over a period of two hours. The eggs were then left to melanize for one hour after removal before rinsing the eggs onto filter paper. The water was allowed to drain prior to sealing the eggs on the filter paper, in an appropriately labelled plastic bag for storage until experiments started. Eggs were stored in the laboratory at ambient temperature during summer months. In experiments conducted during the colder months the eggs were kept in an incubator set at 25°C (humidity was not controlled, but was raised above ambient by placing a beaker of water for evaporation in the incubator).

### Egg metabolic rate measurements

The metabolic rates of sets of 150 eggs were measured every 24 hours up to 8 days old. Eggs were drawn from the larval time-to-hatch selected *An. gambiae* sub-colonies. The total metabolic output of batches of 150 eggs was measured by determining the amount of CO_2_ emitted by the eggs in a closed system using a CO_2_ analyzer. CO_2_ measurements were obtained using an infrared CO_2_ analyzer (LI-CO 6262, Li-Cor, Lincoln, NE, USA) in a similar way to that described by Woods & Singer [26]. Thirty ml glass syringes (Becton Dickson, Franklin Lakes, NJ, USA) with a small hole drilled through the wall of each syringe closest to the top (plunger end) served as respiratory chambers. A set of 150 eggs from either early or late time-to-hatch parents was placed onto a 1 cm^2^ piece of moist filter paper using a very fine paint brush (Herberts and Evans). The eggs on filter paper were then placed into the respiratory chamber. A three-way stopcock was placed between the syringe and the needle, and to prevent leakage, connections were sealed with petroleum jelly. A total of three syringes were used during each experiment. Initially one syringe contained no eggs (control) to obtain a baseline reading and the remaining two syringes contained sets of 150 eggs. Thereafter each syringe served as its own control and one group of early eggs and one group of late eggs was placed into each syringe consecutively so that two or three replicates per sub-colony and per egg batch were measured on the same day. This method was adopted as readings differed slightly between syringes.

Humid CO_2_ free air was obtained by pumping room air through a soda lime scrubber followed by a humidifier. After the eggs had been placed into the syringes they were flushed with the humid CO_2_ free air for five minutes. Once flushing was complete the plunger was depressed to the 20 ml mark, blocking the hole so that the purged air was flushed through the needle. Eggs were left in the sealed CO_2_ free chamber for 1 hr. Air scrubbed of CO_2_ and water using soda lime and magnesium perchlorate, respectively, was drawn through the CO_2_ analyzer at a rate of 100 ml/min. After the 1 hour 5 ml air boluses from each syringe were inserted into the air stream, before the water scrubber. This was done for each syringe in sequence. This process was repeated twice (3 measurements per syringe) and the amount of CO_2_ in the injected samples converted from parts per million (ppm) to ml by integration of the CO_2_ curve and then multiplied by 1000 to give a value in µl. Data were recorded and analysed using DatacanV (Sable systems, Las Vegas, NV, USA). Comparisons of CO_2_ output between syringes as well as between time-to-hatch egg batches were based on two-sample t tests using Statistix 7 (Tallerhasse, USA).

### Observing embryo development in fixed and de-chorinated mosquito eggs

Eggs were collected from mixed age groups of early or late time-to-hatch adults that had received at least two blood meals prior to egg collection. Eggs were stored in distilled water until they reached the developmental age at which viewing was required. Eggs were collected on a strip of filter paper and covered in a few drops of fixative solution: 3.6 M formaldehyde; 0.87 M glacial acetic acid; and 8.5 M absolute ethanol (FAA), for at least 30 minutes. Once eggs had been fixed the filter paper strip with eggs was placed into a 1.5 ml reaction tube and covered with one ml of decalcifying solution. The decalcifying solution was as described by Trpis [27] modified slightly and used to decalcify the chorion so that embryos could be viewed under the microscope. The decalcifying solution contained 0.59 M sodium hypochlorite and 0.35 M glacial acetic acid in distilled water. This successfully fixed the eggs so as to prevent hatching and bleached the chorion so that embryos could be viewed under the microscope. The date the eggs were collected, the age of the eggs in hours or days post oviposition and the cage of adults the eggs were collected from were noted. This ensured that at least three batches of eggs from different cages were collected for viewing. The embryo of each egg was viewed with a Zeiss Stereo Discovery V12 microscope at 150 times magnification. Digital images of embryos were obtained and scale bars inserted. Embryos were compared qualitatively by time post oviposition by qualitatively describing the level of embryo development based on whether segmentation was visible as well as the overall degree of development.

## Results

### Time-to-hatch monitoring

**Figure 1:**
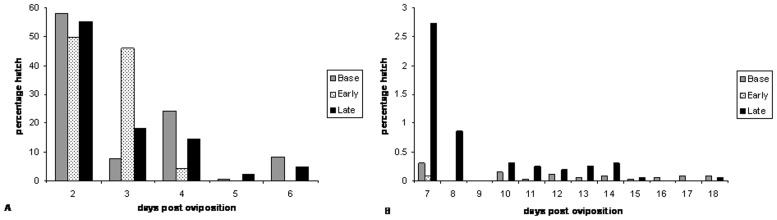
Proportional hatch rate of eggs from the *Anopheles gambiae* baseline colony as well as the early and late time-to-hatch sub-colonies, A) from days two to 6 post oviposition and B) from days 7 to 18 post oviposition.

Five to seven batches of eggs were obtained from each of the time-to-hatch selected sub-colonies as well as from the baseline colony. The sample sizes were 3692, 2381 and 5189 for the early time-to-hatch selected sub-colony, the late time-to-hatch selected sub-colony and the baseline colony respectively. In all cases the majority of the eggs hatched on the second day post-oviposition. The baseline colony then showed less hatching on day 3 post oviposition following which hatching occurred in a staggered fashion with most of the eggs hatching within 6 days post oviposition (Fig. 1A). The last recorded hatching in the baseline colony was on day 18 post-oviposition, as was also the case in the late time-to-hatch selected sub-colony (Fig. 1B) although the numbers were very small (three and one, respectively). The mean times to last hatch were 10.2 (±5.02), 5 (±1.41) and 13.6 (±2.43) for the baseline colony, the early time-to-hatch sub-colony and the late time-to-hatch sub-colony respectively. These differences were statistically significant (ANOVA: F = 10.39, DF = 2, p≤0.01). The primary difference was in a comparison between the early and late time-to-hatch sub-colonies (two sample t-test: t = 7, DF = 1, p<<0.01). There were no significant differences in the mean times to last hatch between the baseline colony and the late time-to-hatch sub-colony (two sample t-test: t = 1.56, DF = 1, p = 0.15) or between the baseline colony and the early time-to-hatch sub-colony (two-sample t-test: t = 2.23, DF = 1, p = 0.08). There were no differences in the total number of eggs that hatched between the baseline and the time-to-hatch selected sub-colonies (means ranged from 58.55–64.05%).

### Egg metabolic rate

**Figure 2:**
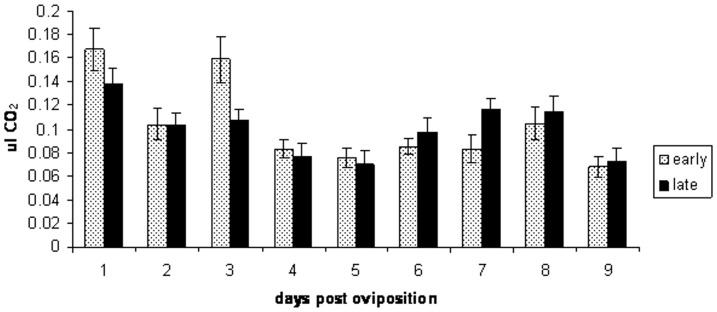
CO_2_ output (µl) of eggs obtained from early and late time-to-hatch selected *Anopheles gambiae* parents by age. Standard error bars are shown.

The amount of CO_2_ produced by age did not differ significantly between the early and late time-to-hatch sub-colonies (ANOVA: p>0.05 for all comparisons, Table 1). In general, egg metabolic rate tended to decrease as the eggs aged up to day 5 post oviposition after which a slight increase in metabolic rate was observed from days 6 to 8, particularly for the eggs from the late-time-to-hatch sub-colony. The highest metabolic rate for both groups of eggs was recorded 24 hours post oviposition (Fig. 2). Based on linear regression, the metabolic output of eggs from the early time-to-hatch sub-colony decreased significantly with age (F = 6.35, DF = 1, p = 0.04; r^2^ = 0.48). The data from the late time-to-hatch colony did not show a significant trend with age (F = 1.20, DF = 1, p = 0.31; r^2^ = 0.15). On day 3 the eggs obtained from early time-to-hatch selected parents produced a significantly higher amount of CO_2_ than the eggs obtained from the late time-to-hatch selected parents (two sample t-test: t = 2.42, DF = 1, p = 0.02) while the reverse was true at day 7 post oviposition (t = −2.06, DF = 1, p<0.05).

**Table 1:** Linear regressions and analysis of variance (ANOVA) indicators obtained from trend analysis of CO_2_ outputs with age by syringe as well as comparisons between the CO_2_ outputs of batches of eggs obtained from the early and late time-to-hatch selected *Anopheles gambiae* sub-colonies.

Regressions	R^2^	P
s2e-age	0.52	*0.02*
s3e-age	0.26	0.15
s2l-age	0.42	0.06
s3l-age	0.03	0.68
sce-age	0.48	*0.04*
scl-age	0.15	0.31

ANOVA	F	P
s2e-s2l	0.14	0.71
s3e-s3l	0.28	0.61
s2e-s3e	0.07	0.79
s2l-s3l	1.72	0.21
sce-scl	0.07	0.79

The degree of freedom for all tests was 1.

*s2  =  syringe 2; s3  = syringe 3; sc  =  syringes combined; e =  eggs from the early time-to-hatch sub-colony; l =  eggs from the late time-to-hatch sub-colony.

### Qualitative embryo development observations

**Figure 3:**
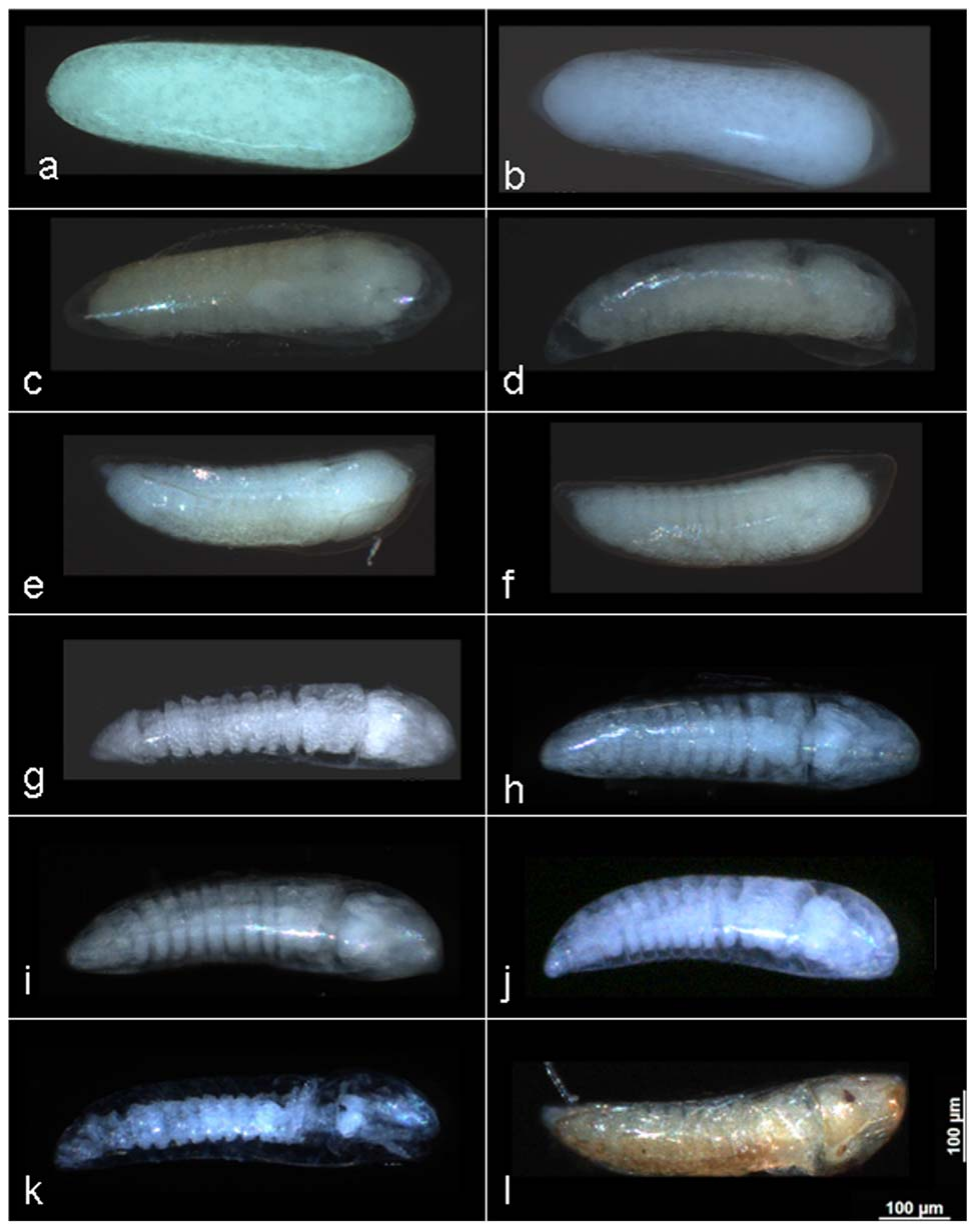
Embryos photographed at different ages and developmental stage post oviposition. a) early 4 hours post oviposition b) late 4 hours post-oviposition c) early 20 hours post oviposition d) late 20 hours post oviposition e) early 24 hours post oviposition f) late 24 hours post oviposition g) early 48 hours post oviposition h) late 48 hours post oviposition i) early 4 days post oviposition j) late 4 days post oviposition k) early 6 days post oviposition l) late 6 days post oviposition.

Approximately 30 images of eggs per age group (1–8 days post oviposition) were obtained from both early and late time-to-hatch selected sub-colonies. Using the degree of definition in embryo segmentation as a visual cue for development, no obvious differences were observed in the rates of embryo development between the eggs from the early and late time-to-hatch sub-colonies (Table 2 and Fig. 3). Most of the embryos from both groups of eggs were fully developed by two to three days post oviposition. Both groups also had eggs that hatched on day two post oviposition. The majority of eggs hatched between days two and four as previously described. Many embryos did not develop. The mean rate to full embryonic development across the ages 24 hours to 8 days was 44.88% in the early time-to-hatch sub-colony and 46.75% in the late time-to-hatch sub-colony.

**Table 2:** Qualitative description of the degree of development of unhatched embryos by age post oviposition for eggs obtained from early and late time-to-hatch selected *Anopheles gambiae* parents.

Days post oviposition	Early time-to-hatch		Late time-to-hatch	
	No. Images taken	Description	No. Images taken	Description
**4–6 hrs**	31	15 white granular with lateral floats often visible. The rest were white-granular with some clearer sections, possibly the start of tissue differentiation Many had a large round tissue mass in the central region.	48	20 were white granular. Most were white-granular on the anterior and posterior ends with a clearer region in the centre of material (only one embryo).
**1 Day**	58	**83%** showed form and evidence of segmentation, often with a gap between the embryo and the shell. Head and body could be defined in most cases.	86	**63%** showed form and segmentation. The rest were granular white or patched white, some with the central tissue mass
**2 Days**	34	**44%** showed clear form and segmentation (most did not fill the whole shell cavity). Hatching had begun.	31	**71%** showed clear form and segmentation or appeared to be developing.
**3 Days**	30	**36.7%** were fully developed taking up the whole shell cavity or close to fully developed	27	**18.5%** were fully developed taking up the whole shell cavity
**4 Days**	31	**58.1%** were fully or very close to fully developed	31	**48.4%** fully or close to fully developed
**5 Days**	27	**48.1%** fully developed or close to fully developed	39	**38.5%** fully or close to fully developed
**6 Days**	30	**20%** were fully developed or close to fully developed	28	**43%** were fully or nearly fully developed
**7 Days**	27	**26%** were fully developed or close to fully developed	31	**58.1%** were fully developed or close to fully developed
**8 Days**	28	**43%** were fully developed or nearly fully developed	33	**36%** were fully or close to fully developed

The total percentages of developed and developing embryos are shown in bold.

## Discussion and Conclusions

The late time-to-hatch selected sub-colony showed a later mean time to last hatch than the early time-to-hatch colony. Under standard insectary conditions, eggs from both time-to-hatch sub-colonies tended to hatch early (four days post-oviposition and earlier) although the late time-to-hatch colony showed a higher proportion of eggs that hatched 5 days post-oviposition and later. In both sub-colonies a very small proportion of eggs hatched up to 18 days post-oviposition.

The ability to delay hatch when conditions for hatching are not immediately suitable is an important adaptive trait in some mosquito species- *Aedes* species in particular [19], [20]. Although dormancy in tropical insects is known, it is perhaps not often considered [28]. The delayed or staggered time-to-hatch described here for the *An. gambiae* baseline and sub-colonies has also been observed in families reared from wild-caught *An. gambiae* females collected in Ghana and the Republic of the Congo [21] as well as from colonised and wild caught mosquitoes from Kenya [18], [19], [20], showing that this trait is not unique to laboratory strains or particular ecological zones although considerable differences are expected to be seen between different populations. Staggered time-to-hatch likely carries significant adaptive significance in *An. gambiae* [21].

The metabolic rates of eggs from the early and late time-to-hatch selected sub-colonies only showed significant variation at three and 7 days post-oviposition. Woods *et al*. [29] showed that metabolic rates in eggs of the moth, *Munduca sexta*, were generally low in the beginning of development and then increased more or less continuously until hatching occurred at the end of development (within 2–3 days). However, Kambule *et al*. [30] showed that the metabolic rate of nondiapause locust eggs continued to increase until the eggs hatched while the metabolic rate of diapause eggs was consistently lower. The highest metabolic rates in the *An. gambiae* eggs were recorded at 24 hours post oviposition which corresponds to the stage in development when embryos undergo a 180° rotation around the longitudinal axis during the germ band retraction phase. Prior to this most of the development has already occurred with intersegmental furrows visible and the ventral aspect of the embryo facing the flattened dorsal side of the shell [31]. Metabolic rates then tended to decrease with time post oviposition in the sub-colonies suggesting that late hatching eggs likely enter a state of diapause prior to hatching.

Wilkenson *et al*. [32] found that 29% and 1% of *An. dirus* eggs survived after 21 days and 92 days respectively on moist filter paper. An experiment by Darrow [33] on *An. quadrimaculatus* eggs showed that eggs removed from water within 9 hours of oviposition could not resist desiccation. However, those removed from water at 10–13 hours post oviposition did develop desiccation tolerance and 27% of these eggs hatched after exposure to 0% RH for 12 hours. Darrow [33] also removed eggs from water at 23.5 hours post oviposition and returned them to water after 24 and 36 hours respectively. The eggs subjected to this treatment hatched approximately 23.5 hours after being returned to water suggesting that embryonic development ceases during desiccation and then continues at the normal rate. In this study the eggs used for egg metabolic rate experiments and monitored for time-to-hatch post oviposition hatched almost immediately after being returned to water. This occurred in all eggs except for 24 hour old eggs, indicating that, similar to [33], eggs can only begin to hatch approximately two days after oviposition when the embryo is fully developed. This suggests that the eggs at ages two days and older were ready to hatch when taken for use in experiments, but that hatch was delayed.

Qualitative embryo development observations were somewhat hampered by the hatching of the majority of eggs between two and four days post oviposition which made the acquisition of unhatched eggs for age groups older than 4 days difficult. As a result many batches of eggs were sampled to acquire adequate sample sizes of late hatching eggs. There were no consistent morphological differences observed between the two groups of eggs in terms of the level of embryo development by age group or between the number of embryos that were fully developed in eggs from the two time-to-hatch selected sub-colonies by age. This may be partly due to the fact that both sub-colonies contain mostly early time-to-hatch eggs, with the late time-to-hatch colony showing a higher proportion of late hatchers. In general, all embryos that developed fully did so within four days post oviposition, supporting the metabolic output data which suggests that late hatching eggs enter a state of diapause once the embryos are fully developed.

Time-to-hatch in Anopheline mosquito eggs is generally believed to be fixed so that it occurs at the completion of embryo development. This is normally at approximately 50 hours old in optimal conditions [33], [34]. However, various factors, temperature in particular, are known to affect that rate of embryo development and hatching [12]. It makes sense that eggs are able to adapt somewhat to different hatching conditions in response to risks and opportunities. Parents are also able to influence hatch timing, so the assumption that hatch timing is relatively fixed is not true (see review by Warkentin, [35]). The current study found that the embryos develop within the first two to four days post oviposition with most hatching immediately and a small proportion delaying hatching. The delayed hatch appears to be accompanied by a fairly constant and reduced metabolic rate in some eggs. As all eggs were exposed to the same environment this phenomenon can not be considered quiescence but rather is assumed to be late embryonic diapause as seen in the Gypsy moth *Lymantria dispar* [36] and some temperate mosquito species [37].

Hatch timing may influence other traits such as insecticide resistance and fitness. Recently Perez and Noriega [38] showed that extended quiescence (eggs induced to hatch after 10 weeks post oviposition instead of after just less than one week for short quiescence) affected performance and reproductive fitness of adult *Aedes aegypti* females and the nutritional status of their progeny via maternal effect. Specifically, the females from the eggs that underwent extended quiescence survived 10% longer, laid more eggs and produced 14% more viable offspring when reared on a sub-optimal diet of 3% sucrose solution. However, the reproductive success of females reared from extended quiescent eggs was dramatically affected by stress in the larval environment in the form of metal contamination. The authors claim that intrapopulation variation in the sensitivity of individuals to environmental cues is what leads to asynchronous hatching and suggest that phenotypic plasticity results as a consequence of pharate (waiting to emerge or hatch) first instar larvae. That variation in sensitivity to environmental stimuli may lead to asynchronous hatching is supported by Ebrahimi *et al* [22] who found that all *An. gambiae* eggs in their experiments required agitation to hatch and that some eggs required more agitation events than others. This is compared to instalment hatching or bet hedging observed in *Aedes* mosquitoes where only a proportion of eggs will hatch in response to a given stimulus [23]. Ebrahimi *et al* [22] point out that this requirement for agitation to induce hatching may be an advantage in mass rearing situations where a large number of synchronously developing mosquitoes are needed. Most studies looking at delayed hatching of *An. gambiae* complex eggs have done so in conjunction with desiccation conditions. Whether the delayed hatching phenotypes observed in this study may be more desiccation tolerant than early hatching phenotypes remains to be investigated.

An association between insecticide susceptibility and staggered time-to-hatch in *An. gambiae* has previously been described [21]. These studies suggest that variation in one trait (such as staggered time-to-hatch) may affect and even enhance variability in other traits that affect reproductive and physiological fitness as well as environmental adaptability. This may occur as a result of resource re-allocation or a as consequence of pleiotropy. It is likely that there are interactions between environmental and genetic factors that influence time-to-hatch in mosquitoes [4], [21], [23]. These factors in turn may influence subsequent life history traits and may even affect behaviour as a consequence of pleiotropy. It would be interesting to determine whether eggs associated with more permanent larval sites such as those of *An. funestus* are more or less likely to hatch as soon as embryos are fully developed without the requirement of a disturbance. Beier *et al* [18] indicate that *An. funestus* is less able to resist desiccation and this may also translate to the ability to delay hatching.

It is concluded that all viable embryos in *An. gambiae* develop to full maturity at the same rate and that a proportion are able to delay hatching. As it has previously been shown that it is possible to at least partially select for late time-to-hatch, this characteristic is likely to involve genetic as well as environmental factors. This study supports several others [11], [18], [19] in which it was also concluded that delayed time-to-hatch in *Anopheles gambiae* is likely an adaptation to maximise reproductive output despite the increased risk of desiccation in an unstable aquatic environment.
